# Biomarkers of Alzheimer’s Disease and Cerebrovascular Disease in Relation to Depressive Symptomatology in Individuals With Subjective Cognitive Decline

**DOI:** 10.1093/gerona/glad216

**Published:** 2023-09-14

**Authors:** Mariola Zapater-Fajarí, Patricia Diaz-Galvan, Nira Cedres, Therese Rydberg Sterner, Lina Rydén, Simona Sacuiu, Margda Waern, Anna Zettergren, Henrik Zetterberg, Kaj Blennow, Silke Kern, Vanesa Hidalgo, Alicia Salvador, Eric Westman, Ingmar Skoog, Daniel Ferreira

**Affiliations:** Division of Clinical Geriatrics, Center for Alzheimer Research, Department of Neurobiology, Care Sciences, and Society, Karolinska Institute, Stockholm, Sweden; Laboratory of Cognitive Social Neuroscience, Department of Psychobiology and IDOCAL, University of Valencia, Valencia, Spain; Division of Clinical Geriatrics, Center for Alzheimer Research, Department of Neurobiology, Care Sciences, and Society, Karolinska Institute, Stockholm, Sweden; Department of Radiology, Mayo Clinic, Rochester, Minnesota, USA; Department of Psychology, Sensory Cognitive Interaction Laboratory (SCI-Lab), Stockholm University, Stockholm, Sweden; Facultad de Ciencias de la Salud, Universidad Fernando Pessoa Canarias, Las Palmas de Gran Canaria, España; Centre for Ageing and Health at The University of Gothenburg, Gothenburg, Sweden; Neuropsychiatric Epidemiology Unit, Department of Psychiatry and Neurochemistry, Institute of Neuroscience and Physiology, Sahlgrenska Academy at The University of Gothenburg, Gothenburg, Sweden; Centre for Ageing and Health at The University of Gothenburg, Gothenburg, Sweden; Neuropsychiatric Epidemiology Unit, Department of Psychiatry and Neurochemistry, Institute of Neuroscience and Physiology, Sahlgrenska Academy at The University of Gothenburg, Gothenburg, Sweden; Division of Clinical Geriatrics, Center for Alzheimer Research, Department of Neurobiology, Care Sciences, and Society, Karolinska Institute, Stockholm, Sweden; Neuropsychiatric Epidemiology Unit, Department of Psychiatry and Neurochemistry, Institute of Neuroscience and Physiology, Sahlgrenska Academy at The University of Gothenburg, Gothenburg, Sweden; Centre for Ageing and Health at The University of Gothenburg, Gothenburg, Sweden; Region Västra Götaland, Sahlgrenska University Hospital, Psychosis Department, Gothenburg, Sweden; Centre for Ageing and Health at The University of Gothenburg, Gothenburg, Sweden; Neuropsychiatric Epidemiology Unit, Department of Psychiatry and Neurochemistry, Institute of Neuroscience and Physiology, Sahlgrenska Academy at The University of Gothenburg, Gothenburg, Sweden; Neuropsychiatric Epidemiology Unit, Department of Psychiatry and Neurochemistry, Institute of Neuroscience and Physiology, Sahlgrenska Academy at The University of Gothenburg, Gothenburg, Sweden; Clinical Neurochemistry Laboratory, Sahlgrenska University Hospital, Mölndal, Sweden; Neuropsychiatric Epidemiology Unit, Department of Psychiatry and Neurochemistry, Institute of Neuroscience and Physiology, Sahlgrenska Academy at The University of Gothenburg, Gothenburg, Sweden; Clinical Neurochemistry Laboratory, Sahlgrenska University Hospital, Mölndal, Sweden; Neuropsychiatric Epidemiology Unit, Department of Psychiatry and Neurochemistry, Institute of Neuroscience and Physiology, Sahlgrenska Academy at The University of Gothenburg, Gothenburg, Sweden; Region Västra Götaland, Sahlgrenska University Hospital, Clinic for Psychiatry, Cognition and Old Age Psychiatry, Gothenburg, Sweden; Laboratory of Cognitive Social Neuroscience, Department of Psychobiology and IDOCAL, University of Valencia, Valencia, Spain; IIS Aragón, Department of Psychology and Sociology, Area of Psychobiology, University of Zaragoza, Teruel, Spain; Laboratory of Cognitive Social Neuroscience, Department of Psychobiology and IDOCAL, University of Valencia, Valencia, Spain; Spanish National Network for Research in Mental Health CIBERSAM, Madrid, Spain; Division of Clinical Geriatrics, Center for Alzheimer Research, Department of Neurobiology, Care Sciences, and Society, Karolinska Institute, Stockholm, Sweden; Department of Neuroimaging, Centre for Neuroimaging Sciences, Institute of Psychiatry, Psychology and Neuroscience, King’s College London, London, UK; Neuropsychiatric Epidemiology Unit, Department of Psychiatry and Neurochemistry, Institute of Neuroscience and Physiology, Sahlgrenska Academy at The University of Gothenburg, Gothenburg, Sweden; Region Västra Götaland, Sahlgrenska University Hospital, Clinic for Psychiatry, Cognition and Old Age Psychiatry, Gothenburg, Sweden; Division of Clinical Geriatrics, Center for Alzheimer Research, Department of Neurobiology, Care Sciences, and Society, Karolinska Institute, Stockholm, Sweden; Facultad de Ciencias de la Salud, Universidad Fernando Pessoa Canarias, Las Palmas de Gran Canaria, España; (Medical Sciences Section)

**Keywords:** Amyloid-beta, Cerebrovascular disease, Depressive symptomatology, Phosphorylated tau, Subjective cognitive decline

## Abstract

**Background:**

Subjective cognitive decline (SCD) has gained recent interest as a potential harbinger of neurodegenerative diseases such as Alzheimer’s disease (AD) and cerebrovascular disease (CVD). In addition, SCD can be related to depressive symptomatology. However, the association between AD and CVD biomarkers, depressive symptomatology, and SCD is still unclear. We investigated the association of AD and CVD biomarkers and depressive symptomatology with SCD in individuals with subjective memory complaints (SCD-memory group) and individuals with subjective concentration complaints (SCD-concentration group).

**Methods:**

We recruited a population-based cohort of 217 individuals (all aged 70 years, 53% female participants, 119 SCD-memory individuals, 23 SCD-concentration individuals, and 89 controls). AD and CVD were assessed through cerebrospinal fluid levels of the Aβ42/40 ratio and phosphorylated tau, and white matter signal abnormalities on magnetic resonance imaging, respectively. Associations between biomarkers, depressive symptomatology, and SCD were tested via logistic regression and correlation analyses.

**Results:**

We found a significant association between depressive symptomatology with SCD-memory and SCD-concentration. Depressive symptomatology was not associated with AD and CVD biomarkers. Both the phosphorylated tau biomarker and depressive symptomatology predicted SCD-memory, and the Aβ42/40 ratio and depressive symptomatology predicted SCD-concentration.

**Conclusions:**

The role of depressive symptomatology in SCD may differ depending on the stage within the spectrum of preclinical AD (as determined by amyloid-beta and tau positivity), and does not seem to reflect AD pathology. Our findings contribute to the emerging field of subclinical depressive symptomatology in SCD and clarify the association of different types of subjective complaints with distinct syndromic and biomarker profiles.

Subjective cognitive decline (SCD) has been suggested as one of the first signs of Alzheimer’s disease (AD) (1). Previous studies showed a significant association between SCD and biomarkers of AD pathology, including amyloid-beta (Aβ) and tau biomarkers (2,3). In addition, SCD may also be associated with other brain pathologies such as cerebrovascular disease (CVD) (4–8). Hence, SCD is gaining interest as an early marker of various brain pathologies, which can be diagnosed before the emergence of objective cognitive impairment.

A current topic of discussion is that SCD may reflect not only brain pathologies but also other nonpathological conditions such as depressive symptomatology (7,9). As a consequence, the leading international SCD-Initiative (SCD-I) working group has recently made a call for a better understanding of the role of depressive symptomatology in SCD (10). Several studies have shown a strong association between SCD and depressive symptomatology (5,9). The main difficulty lies in understanding whether depressive symptomatology found in SCD individuals is really connected to brain pathology (4,11,12). For instance, depression correlates with AD biomarkers in the absence of cognitive impairment (11,13), and AD pathology predicts an increase in depressive symptomatology over time (14). Similarly, CVD is known to affect neural connections leading to depressive symptomatology (12). However, the association between SCD, depressive symptomatology, and AD and CVD biomarkers is not completely understood (10). Another difficulty is that clinical depression is an exclusion criterion for the diagnosis of SCD (1), but how to approach subclinical depressive symptomatology in SCD is still debated (10).

AD pathology (amyloidosis and tau neurofibrillary tangles) can be assessed in vivo through cerebrospinal fluid (CSF) biomarkers such as the amyloid-beta 42/40 (Aβ 42/40) ratio and phosphorylated tau (p-tau) (15). Amyloidosis is thought to be the initiating event of the Alzheimer’s pathological cascade, which would be followed by tau pathology (16,17). SCD has been related to the preclinical stage of AD (10), demonstrating early positivity in AD biomarkers in the absence of overt cognitive impairment (18). CVD can be measured with magnetic resonance imaging (MRI) (19), for example in the form of white matter signal abnormalities (WMSA) on T1-weighted images (white matter hypointensities) and on T2-weighted or fluid-attenuated inversion recovery (FLAIR) images (white matter hyperintensities).

In a previous study, we demonstrated that SCD is independently associated with both subclinical depressive symptomatology and CVD (7), but depressive symptomatology was not associated with CVD and we could not test for associations with Aβ and tau biomarkers. Hence, the question of whether depressive symptomatology in SCD is a symptom of brain pathologies remains partially unanswered. The primary aim of the current study was to investigate the role of depressive symptomatology and biomarkers of brain pathology (CVD, Aβ 42/40, and p-tau) in SCD. We hypothesized that SCD would be related to both depressive symptomatology and biomarkers of AD and CVD (7,8,13). We also wanted to test whether depressive symptomatology found in SCD is associated with brain pathology. We hypothesized that depressive symptomatology would be related to biomarkers of AD and CVD (11–13). Additionally, a recent publication showed that different complaints are associated with different MRI-based biomarker profiles and depressive symptomatology (20). However, there is a need to confirm this finding in independent cohorts that also include CSF biomarkers of AD. Some studies have already made the distinction between memory and concentration complaints (21,22). Therefore, our secondary aim was to investigate memory and concentration complaints separately in relation to depressive symptomatology and biomarkers of AD and CVD. Because memory impairment is a core symptom of AD and difficulties in concentration are common in individuals with high CVD burden, we hypothesized that subjective memory complaints would be more strongly associated with AD biomarkers, and concentration complaints would be more strongly associated with CVD biomarkers (20,22). The association of depressive symptomatology with SCD is well known, so we hypothesized that both memory and concentration complaints would be associated with depressive symptomatology.

## Materials and Methods

### Participants

Data were derived from the 2014–16 examinations of the Gothenburg H70 Birth Cohort 1944 Studies. A total of 1 203 individuals participated in the study. All examinations and procedures have been described previously (23). The initial sample was composed of 297 participants, who received an MRI scan and a CSF lumbar puncture (LP). Inclusion criteria were in concordance with the international SCD-I working group (1):

1) Normal cognition, in this study established in 2 steps: First, dementia was excluded based on a clinical diagnosis of dementia according to the DSM-III-R criteria, a mini-mental state examination (MMSE) score <24, or a Clinical Dementia Rating score >0.5. Second, mild cognitive impairment (MCI) was excluded based on comprehensive neuropsychological assessment and age-, sex-, and education-adjusted normative data. Following recommendations by Jak et al. (24) and Molinuevo et al. (25), individuals were classified as MCI if at least 1 of the following 2 criteria were met: (Criterion 1) performance below the 16th percentile in 2 measures within at least 1 of the following cognitive domains: Memory, represented by Thurstone’s Picture Memory, 10-word list, and remembering 12 objects; Verbal fluency represented by a semantic fluency task (animals); Speed/ executive function, represented by Digit Span Forward and Backward test, and the Figure Logic (SRB2) of the Figure Logic of the Synonyms, Reasoning, and Block Design Test; and Visuospatial ability represented by Block Design (Koh’s Block Test); (Criterion 2) performance below the 16th percentile in 3 independent tests of 3 out 4 cognitive domains studied. When Criterion 1 could not be met because the domain was evaluated with 1 test, Criterion 2 was spent. The 16th percentile criterion was favored instead of the >1SD criterion by Jak et al. (24) and Molinuevo et al. (25) because of the asymmetrical distribution of several neuropsychological tests in our cohort;
2) No large infarcts or tumors on brain MRI and no history of stroke or transient ischemic attack, according to a neuroradiologist;
3) No medical history of psychiatric (e.g., major depression) or neurological disorders, systemic diseases, or head trauma, nor intake of antidepressants; and
4) No history of substance abuse based on clinical interview and no Alcohol Use Disorder Identification Test score >20 (26).

Among 297 participants in the initial sample, 80 were excluded after applying our inclusion criteria. One further participant was excluded due to missing data in the semistructured Comprehensive Psychopathological Rating Scale (CPRS) (27). The final sample was composed of 217 participants. The process of participant selection and the reason for exclusions are shown in Supplementary Figure 1.

### Subjective Cognitive Decline

SCD was assessed using 2 different questions from the semistructured CPRS (27) that refer to subjective memory and concentration complaints experienced during the last month. These questions are rated on a 7-point Likert scale according to intensity, frequency, and degree of inability produced (Supplementary Table 1). The range is from 0 (no difficulties) to 6 (severe difficulties), allowing for intermediate scores. Scores 0 and 1 represent no difficulties or difficulties within the normal range, whereas scores ≥2 reflect an increasing degree of complaint. This type of response in a Likert scale has been suggested as the better choice to measure change over time by the SCD-I and is commonly used in studies of the world-leading initiative (80% of the studies) (28). To note, the concentration item further extends the study of SCD so far primarily focused on memory complaints, strengthening the notion about different phenotypes associated with different complaints (20). Furthermore, our 2 questions for memory and concentration provide a simplified assessment of 2 distinct cognitive domains in validated scales for SCD such as the Everyday Cognition scale (ECog) (29), which is recommended by the SCD-I. Supplementary Table 2 shows the correspondence between our 2 questions for memory and concentration and the respective items of ECog. Based on clinical experience and clinimetric considerations about the CPRS, the presence of a complaint was defined by the cutoff point of ≥2 on the concentration and memory complaints items. Indeed, the CPRS itself proposes that scores <2 represent no difficulties or difficulties within the normal range, whereas scores 2 or more reflect some degree of complaint. Based on this criterion, individuals were classified into people with SCD in memory (SCD-memory group) or concentration (SCD-concentration group) if they scored ≥2 in these items, respectively. Individuals who scored <2 on both memory and concentration complaints items were classified as controls. We favored this dichotomous classification (SCD vs controls) instead of the continuous form of the items due to the study aims and the nature of our statistical approach (see later).

### Depressive Symptomatology

The Montgomery-*Åsberg Depression Rating Scale* (MADRS) (30) was derived from the CPRS and used to assess the overall burden of depressive symptoms. The MADRS is a 10-item scale scored on a 7-point Likert scale from 0 (no symptoms) to 6 (severe symptoms), giving a total score from 0 to 60 (MADRS-10). We used the total MADRS-10 score to characterize the cohort (clinical cut points are available for the MADRS-10 score). However, our main statistical analyses were performed on the MADRS score with the concentration item excluded (MADRS-9, based on 9 items), to avoid circularity, because the concentration item of the CPRS defines the SCD-concentration group. In addition, as explained in the “Participants” section, none of the participants had a clinical diagnosis of major depression nor were they under treatment for depression, in agreement with the current diagnostic criteria of SCD (1).

### MRI Acquisition, Image Processing, and Biomarkers of CVD

Participants were scanned using a 3.0T Philips Achieva system (Philips Medical Systems, Netherlands). It used a 3D T1-weighted Turbo Field Echo sequence (repetition time = 7.2 milliseconds, echo time = 3.2 milliseconds, flip angle = 9°, number of slices = 160, matrix size = 250 × 250 mm, field of view = 256 × 256 mm, and slice thickness = 1.0 mm) and a 3D FLAIR sequence (repetition time= 4 800 milliseconds, echo time = 280 milliseconds, inversion time = 1 650 milliseconds, flip angle = 90°, number of slices = 140, matrix size=250 × 237 mm, field of view = 250 × 250 mm, and slice thickness = 2.0 mm) for estimations of hypointense and hyperintense WMSA, respectively. Data management and image processing were done with our database system the HiveDB (31).

Both hypointense and hyperintense WMSA were used as biomarkers of CVD. Although there is a strong correlation between hypointense and hyperintense WMSA (32,33), they may reflect different underlying pathologies. Hypointense WMSA has been suggested to reflect poorer white matter integrity and more chronic white matter damage than hyperintense WMSA (33). In contrast, hyperintense WMSA may reflect a mix of white matter damage, peri-inflammatory processes, and other pathologies related to increased blood–brain barrier permeability (34). Given that age-related/ neurodegenerative CVD is usually insidious and chronic rather than acute, in this study, we selected hypointense WMSA as a better proxy of CVD in our population, but reported hyperintense WMSA in supporting information for completeness of information.

Hypointense WMSA were automatically segmented with FreeSurfer 6.0.0 and hyperintense WMSA were automatically segmented with the lesion segmentation tool (LST) 2.0.15. Briefly, the T1-weighted images were processed with the FreeSurfer 6.0.0 image analyses suite (http://surfer.nmr.mgh.harvard.edu/). FreeSurfer detects white matter hypointensities and automatically labels them using a probabilistic procedure (35). This procedure is sensitive in measuring white matter damage both in healthy individuals and in patients with AD (36,37). LST is an open-source segmentation toolbox in the Statistical Parametric Mapping (SPM) software (https://www.fil.ion.ucl.ac.uk/spm/), which uses a lesion prediction algorithm based on FLAIR hyperintensities that builds a lesion probability map for each individual. WMSA volumes in millimeters (ml) were adjusted by the total intracranial volume (TIV) obtained from FreeSurfer. This adjustment was performed by dividing the WMSA volume by the TIV of each participant (38), and TIV-adjusted WMSA measures were used for statistical analyses. Following Cedres et al. (32), we classified WMSA into low and high WMSA burden with a cutoff value of 0.00321 for hypointense WMSA, which resembles low and high WMSA burden in the Fazekas visual rating scale (39). These 2 variables were treated continuously in the analyses but were categorized as high and low to describe the degree of pathology for the characterization of the sample.

### CSF Biomarkers and *APOE*-ε4 Genotype

Cerebrospinal fluid (CSF) samples were collected in the morning through LP in L3/L4 or L4/L5 interspaces. A total of 10 ml of CSF was collected in a polypropylene tube and immediately transported to the laboratory and centrifuged at 1 800*g* at 20° C. The supernatant was gently mixed to avoid possible gradient effects, aliquoted in polypropylene tubes, and stored at −70° C. CSF phosphorylated tau at threonine 181 (p-tau) concentrations were determined using sandwich enzyme-linked immunosorbent assay (INNOTEST htau Ag and PHOSPHO_TAU (181P; Fujirebio, Ghent, Belgium) (40). We used the CSF Aβ42/40 ratio as a biomarker of amyloid-beta pathology. The CSF Aβ42/40 ratio was obtained using the V-PLEX Aβ peptide Panel 1 (6E10) kit (Meso Scale Discovery, Rockville, MD) (41). This variable was treated continuously in the main analyses. To characterize the sample, following Samuelsson et al. (42) we classified CSF p-tau and Aβ42/40 values into positive and negative using the following cutoff values: ≥80 pg/mL for p-tau and ≤0.082 for Aβ42/40. The *APOE*-ε4 allele was determined using the KASPar PCR SNP genotyping system (LGC Genomics, Hoddesdon, Herts, UK) as described by Skoog et al. (43) To characterize the sample, participants were classified as *APOE-*ε4 carriers if they had at least 1 ε4 allele.

### Statistical Analyses

Box-Cox transformations were performed when continuous variables did not follow the normal distribution (44). First, we conducted univariate analyses consisting of Student *t* tests and Pearson’s Chi-square tests. We tested for differences between individuals with subjective complaints in memory and controls (SCD-memory vs controls) across sociodemographic variables, MMSE, *APOE* ε4, depressive symptom scores, CSF p-tau, CSF Aβ42/40, and WMSA. The same analyses were also performed for the comparison between individuals with subjective complaints in concentration and controls (SCD-concentration vs controls). Effect sizes were calculated using Cohen’s *d* for continuous variables ion *t* tests, and using phi (φ) for nominal variables on Chi-square (χ^2^) tests. For Cohen’s *d* 0.20, 0.50, and 0.80 and for φ 0.1, 0.3, and 0.5 represent small, medium, and large effect sizes, respectively. Second, we conducted a multivariable analysis consisting of multiple binary logistic regression models. These models were used to further investigate partial associations of depressive symptom scores, CSF p-tau, CSF Aβ42/40, and WMSA (predictors) with SCD-memory versus controls or SCD-concentration versus controls (outcome variables), in 2 separate models. We report models for hypointense WMSA in the main text and models for hyperintense WMSA in supporting information. Finally, we approximated the question of whether depressive symptomatology is a symptom of brain pathology through correlations of depressive symptom scores with WMSA, CSF p-tau, and CSF Aβ42/40. All statistical analyses were carried out using SPSS v.26 (IBM Statistics, Chicago, IL, USA). All *p* values were 2-tailed and the level of significance was set at *p* < .05.

## Results

### Cohort Description

Cohort characteristics are shown in Table 1. Regarding biomarkers, 32% of the participants were CSF Aβ42/40-positive, 6% were p-tau-positive, and 15% had a high hypointense WMSA burden. Total MADRS-10 scores ranged from 0 to 20 (mean = 2.97, *SD* = 3.66). Hence, consistent with the SCD-I criteria, virtually all the participants had depressive symptom scores within the normal range when using clinical cut points for the MADRS (44), whereas 6 participants had mild depressive symptomatology, and 1 participant had moderate depressive symptomatology. Total MADRS-9 scores used in the analyses ranged from 0 to 18 (mean = 2.67, *SD* = 3.43). The distributions of total MADRS-10 and MADRS-9 scores are shown in Supplementary Figure 2.

**Table 1.** Demographic and Clinical Characteristics of the Total Sample and by SCD-Memory and SCD-Concentration Groups

						SCD-memory vs control group			SCD-concentration vs control group		
	Total sample (*n* = 217)	Min-Max.	SCD-memory group (*n* = 119)	SCD-concentration group (*n* = 23)	Control group (*n* = 89)	*t*/χ^2^	*p* Value	*d*/φ	*t*/χ^2^	*p* Value	*d*/φ
Age (y)	70.54 (0.26)	69.71-71.92	70.55 (0.24)	70.56 (0.18)	70.54 (0.30)	−0.22	.827	−0.03	−0.283	.777	−0.07
Sex (% women)	52.5		47.9	56.5	57.3	1.804	.179	0.10	0.005	.946	0.01
Years of education	13.30 (4.09)	7-35	12.99 (3.67)	13.26 (4.05)	13.65 (4.70)	1.13	.261	0.16	0.361	.719	0.09
*APOE* ε4 (%)	32		29	55	33	0.136	.712	−0.03	3.652	.056	0.18
MMSE (total score)	29.19 (1.07)	25-30	29.16 (1.16)	28.96 (1.29)	29.17 (.97)	0.058	.954	0.01	0.870	.386	0.20
Depressive symptomatology (MADRS-9)	2.67 (3.43)	0-18	3.04 (3.85)	4.39 (4.95)	2.034 (2.61)	−2.25	.026	−0.30	−2.205	.037	−0.73
P-tau levels (pg/L)	49.38 (17.19)	18-128	51.41 (18.12)	50.26 (17.06)	47.27(16.36)	−1.70	.091	−0.24	−0.775	.440	−0.20
P-tau (% positive)	6	—	7	9	6	0.106	.745	0.02	0.295	.587	0.05
Aβ 42/40 ratio	0.087 (0.022)	0.026-0.121	0.086 (0.023)	0.070 (0.024)	0.089 (0.019)	0.968	.334	0.06	3.641	.001	0.86
Aβ 42/40 (% positive)	32	—	33	61	26	1.062	.303	0.07	9.899	.002	0.30
Hypointense WMSA (ICV-corrected volumes)	0.0023 (0.0026)	0.0004-0.022	0.0023 (0.0024)	0.0025 (0.0033)	0.0022 (0.0029)	−0.136	.892	−0.13	−0.387	.699	−0.13
Hypointense WMSA (high burden) (%)	15	—	16	13	12	0.537	.464	0.05	0.008	.930	0.01

*Notes*: *APOE*-ε4 =participants with at least 1 *APOE*-ε4 allele; Aβ 42/40 = Amyloid-beta 42/40 ratio; MADRS-9 = The Montgomery-Åsberg Depression Rating Scale with the concentration item excluded; MMSE= mini-mental state examination; p-tau= phosphorylated tau; SCD-memory = subjective cognitive decline in memory; SCD-concentration = subjective cognitive decline in concentration; WMSA= White matter signal abnormalities; χ^2^ = Chi-square; *d* = Cohen’s *d*; *φ* =phi.

Data represent means (standard deviation) except for sex, *APOE*-ε4, and the dichotomized version of biomarkers, where percentage is shown. *p* values are shown for the differences between SCD-memory and control group, and between SCD-concentration and control group. Analyses involving years of education, Aβ 42/40, and *APOE*-ε4 were performed on *n* = 216, *n* = 216, and *n* = 213 individuals, respectively.

Regarding subjective cognitive complaints, 119 (54.8%) participants endorsed subjective complaints in memory and were thus classified into the SCD-memory group. A total of 23 participants (10.6%) reported subjective complaints in concentration and were thus classified into the SCD-concentration group. A total of 89 participants (41%) did not have any subjective complaints in memory or concentration and were thus classified into the control group. There was no association between SCD-memory and SCD-concentration groups (χ^2^ = .378; *p* = .539).

**Figure 1.**
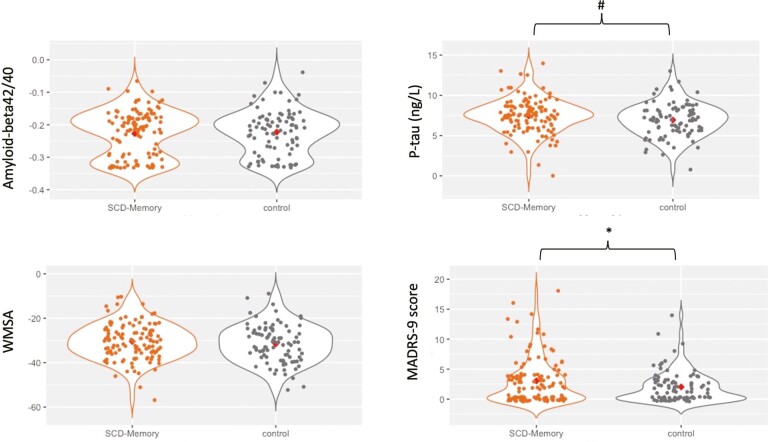
Aβ 42/40, p-tau, hypointense WMSA, and MADRS-9 scores in SCD-memory and control groups. Violin plots where observations and data distribution are represented. Red diamonds represent median values for SCD-memory and control groups. *p* < .05* (*p* = .026 for MADRS-9), # = trend towards significant differences (*p* = .091 for p-tau). Aβ 42/40 = Amyloid-beta 42/40 ratio; MADRS-9= The Montgomery-Åsberg Depression Rating Scale with the concentration item excluded; p-tau= phosphorylated tau; SCD-memory = subjective cognitive decline in memory.

**Figure 2.**
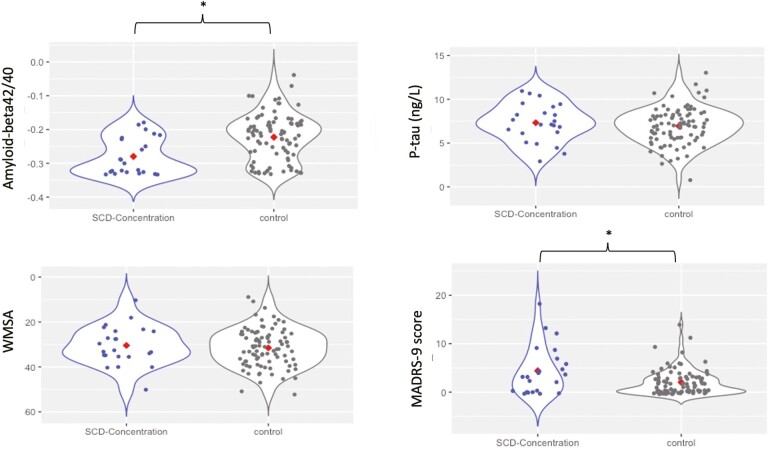
Aβ 42/40, p-tau, hypointense WMSA, and MADRS-9 scores in SCD-concentration and control groups. Violin plots where observations and data distribution are represented. Red diamonds represent median values for SCD-concentration and control group. *p* < .05* (*p* = .001 for Aβ 42/40 and *p* = .037 for MADRS-9). Aβ 42/40 = Amyloid-beta 42/40 ratio; MADRS-9 = The Montgomery-Åsberg Depression Rating Scale with the concentration item excluded; p-tau = phosphorylated tau; SCD-concentration = subjective cognitive decline in concentration.

We found no significant differences in age, sex, years of education, and MMSE between the SCD-memory group and controls, nor between the SCD-concentration group and controls (all *p* > .05). Participants in the SCD-memory group had significantly higher MADRS-9 scores (*p* = .026) and showed a tendency to have higher CSF p-tau levels (*p* = .091), as compared with the control group. The SCD-concentration group had a significantly higher frequency of CSF Aβ42/40-positive individuals (*p* = .002), had lower levels of CSF Aβ42/40 (*p* = .001), had higher MADRS-9 scores (*p* = .037), and showed a tendency to have a higher frequency of *APOE*-ε4 carriers (*p* = .056) than the control group (Table 1). Figures 1 and 2 show the differences in CSF Aβ42/40, p-tau, and MADRS-9 scores between SCD-memory and SCD-concentration groups and the control group.

### Logistic Regression Analyses

#### Partial association of CSF biomarkers, WMSA, and depressive symptomatology with SCD-memory

Binary logistic regression was conducted including SCD-memory group as the criterion variable (SCD-memory vs controls), and MADRS-9, CSF Aβ42/40, CSF p-tau, and hypointense WMSA as the predictors. The model was significant (*p* = .014) showing that higher MADRS-9 scores (*p* = .025, OR = 1.114) and p-tau levels (*p* = .053, OR = 1.142) predicted SCD-memory group. In contrast, CSF Aβ42/40 (*p* =.969, OR = 0.922) and hypointense WMSA (*p* =.265, OR = 1.019) were not associated with SCD-memory group (Table 2). We repeated the same model with hyperintense WMSA instead of hypointense WMSA and the results were similar (see Supplementary Materials).

**Table 2.** Logistic Regression Models

Dependent variable: SCD-memory group (SCD-memory coded as 1, controls as 0)					
χ^2^(2) = 8.550	*R* ^2^ = 0.054 (Nagelkerke)			*p* = .014	
	Beta	Wald	*SE*	*p* Value	OR
MADRS-9	0.108	5.042	0.048	.025	1.114
p-Tau	0.133	3.744	0.069	.053	1.142
Aβ 42/40	−0.082	0.002	2.100	.969	0.922
Hypointense WMSA	0.019	1.242	0.017	.265	1.019

Dependent variable: SCD-concentration group (SCD-concentration coded as 1, controls as 0)					
χ^2^(2) = 21.422	*R* ^2^ = 0.274 (Nagelkerke)			*p* < .001	
	Beta	Wald	*SE*	*p* Value	OR
Aβ 42/40	−15.302	10.813	4.653	.001	0.000
MADRS-9	0.201	7.045	0.076	.008	1.223
p-tau	0.010	0.006	0.131	.941	1.010
Hypointense WMSA	0.032	1.114	0.030	.291	1.032

*Note*: Aβ 42/40 = Amyloid-beta 42/40 ratio; MADRS-9 = The Montgomery-Åsberg Depression Rating Scale with the concentration item excluded; OR = odds ratio; p-tau = phosphorylated tau; SCD-memory= subjective cognitive decline in Memory; SCD-concentration= subjective cognitive decline in concentration; WMSA= White matter signal abnormalities; χ^2^ = Chi-square; *d* = Cohen’s *d*; *φ* =phi.

#### Partial association of CSF biomarkers, WMSA, and depressive symptomatology with SCD-concentration

We performed similar models for the SCD-concentration group. SCD-concentration group was included as the criterion variable (SCD-concentration vs controls), and MADRS-9, CSF Aβ42/40, and CSF p-tau and hypointense WMSA were included as the predictors. The model was significant (*p* < .001). CSF Aβ42/40 (*p* = .001, OR = 0.000) was the main predictor of SCD-concentration group, followed by MADRS-9 (*p* = .008, OR = 1.223). The SCD-concentration group had a lower CSF Aβ42/40 ratio and higher MADRS-9 scores than the control group. In contrast, p-tau (*p* = .941, OR = 1.010) and hypointense WMSA (*p* =.291, OR = 1.032) were not associated with SCD-concentration group (Table 2). When performing the same model with hyperintense WMSA instead of hypointense WMSA, the results were similar (see Supplementary Materials).

### Correlations of Depressive Symptomatology With Biomarkers of Brain Pathology

We did not find any significant correlation between MADRS-9 scores and p-tau, Aβ42/40, or WMSA biomarkers (all *p* > .05; Table 3).

**Table 3.** Correlations Between Depressive Symptomatology and Biomarkers

	Aβ 42/40	Hypointense WMSA	Hyperintense WMSA	MADRS-9
p-tau	−0.119	−0.082	−0.055	-.087
Aβ 42/40		0.029	0.011	-.070
Hypointense WMSA			0.863*	-.015
Hyperintense WMSA				-.051

*Notes*: Aβ 42/40 = Amyloid-beta 42/40 ratio; MADRS-9 = The Montgomery-Åsberg Depression Rating Scale with the concentration item excluded; p-tau= phosphorylated tau; WMSA= White matter signal abnormalities.

Analyses for Aβ 42/40 based on *n* = 216.

*p < .001.

## Discussion

The primary aim of this study was to investigate the association between SCD, depressive symptomatology, and biomarkers of brain pathology (Aβ42/40, p-tau, and WMSA). Additionally, we investigated whether depressive symptomatology is associated with biomarkers of brain pathologies or is rather independent of the underlying pathological process. We extended the research in previous studies by investigating associations between SCD, depressive symptomatology, and biomarkers of AD and CVD pathologies in the same sample of cognitively unimpaired older adults, and by reporting the findings for 2 common subjective complaints separately, that is, memory and concentration complaints.

There has been an intense discussion about the potential confounding effect of depressive symptomatology in SCD (7,9). Major depression is an exclusion criterion for SCD (1), but subclinical depressive symptomatology is recognized and further research on its role in SCD has recently been promoted by the SCD-I workgroup (10). However, very little is known about what should be the exact threshold to exclude depression in SCD studies and how this type of symptomatology should be assessed, while the common approach is to exclude clinical depression. Instead, subclinical depressive symptomatology can be included in SCD studies and the current need is to clarify its role in SCD (10,25). Our main finding confirmed the well-known association between subclinical depressive symptomatology and SCD. In addition, we demonstrated that the association between depressive symptomatology and SCD was independent of AD and CVD biomarkers. Our logistic regression analyses showed that depressive symptom scores do not seem to influence the association of amyloid-beta, tau, and WMSA biomarkers with SCD. Although this association has not been extensively investigated, some previous studies also showed that depressive symptomatology can co-exist with brain pathologies (3,7,8).

Depressive symptom scores did not correlate with AD and CVD biomarkers in our study. Similarly, Diaz-Galvan et al. (7) did not find any significant association between depressive symptomatology and a CVD biomarker. However, other studies did find significant associations between depression and AD biomarkers (45) and CVD (8). These contradictory results may be due to the fact that in the articles reviewed by Harrington et al. (45) participants had higher levels of depression than in our sample, mostly representing major depression or dysthymia; and by Minett et al. (8) depression was operationalized as a history of major depression. Hence, in these previous studies, the measures of depression reflected clinical depression. In contrast, our cohort primarily reflects variability in the subthreshold spectrum of depressive symptomatology (subclinical depressive symptoms). Only 7 participants had scores above the clinical cut point for depression: 6 were within the mild range and 1 was within the moderate range of depressive symptomatology. As per exclusion criteria, our participants did not had a clinical diagnosis of major depression nor were they under treatment for depression, in agreement with the current diagnostic criteria of SCD (1). Altogether, our results suggest that subclinical depressive symptomatology in our SCD individuals did not reflect AD or CVD pathologies.

We found different associations depending on the type of subjective complaint. Concentration complaints were mainly associated with the amyloid-beta biomarker, followed by depressive symptom scores. In contrast, memory complaints were mainly associated with depressive symptoms score, followed by the tau biomarker. To the best of our knowledge, only 2 studies have addressed the distinction between subjective complaints of memory and concentration (21,22). Both studies partially agree with our results by showing that more depressive symptomatology was associated with memory and concentration complaints, although with a stronger association with concentration complaints (21,22). However, these previous studies did not exclude concentration items from the depression scales. In contrast, our current study did exclude the concentration item from the depression score to avoid circularity. This could influence the strength of the associations because difficulties in concentration are frequent in people with depressive symptomatology.

Regarding AD biomarkers, we found that concentration complaints were associated with the amyloid-beta biomarker, while memory complaints were associated with the tau biomarker. Despite not specifically assessing concentration complaints, Amariglio et al. (2) found an association of nonmemory subjective complaints with their amyloid-beta biomarker. In contrast, Grambaite et al. (21) reported that memory and concentration complaints were not associated with amyloid-beta, p-tau, or total tau CSF biomarkers in individuals with memory complaints (21). The lack of a significant association by Grambaite et al. (21) could be related to the small sample size (*N* = 23) and/or inclusion of a younger cohort (mean age, 58.8 years) since AD biomarker positivity increases with age (45). Additionally, several studies that did not differentiate the type of complaint but investigated an SCD group, found significant associations with AD biomarkers (2,3,18).

The AD continuum of the NIA-AA classification system (46) places SCD at the latest stage of the preclinical phase, also known as the transitional stage (Stage 2). In that stage, ­amyloid-beta and tau biomarkers are positive, but there is no formal evidence of objective cognitive impairment (10). Our differential associations of concentration and memory complaints with amyloid-beta and tau biomarkers could be interpreted using the hypothetical model of dynamic biomarkers proposed by Jack et al. (16,17). In that model, amyloid-beta positivity precedes positivity in tau biomarkers. Following this hypothesis, our results could be interpreted as concentration complaints being an early sign of amyloid-beta pathology, hence reflecting an AD pathological change (47). In contrast, memory complaints would be a sign of tau pathology, hence reflecting AD pathology and signifying a more developed disease status closer to the clinical transition to MCI (47). This is also an interesting finding when it comes to interpreting the role of depressive symptomatology in SCD. In our cohort, depressive symptomatology seems to be an important factor at the end of the SCD continuum, showing a significant association with memory complaints, which in turn reflect tau pathology in our cohort. However, in the context of ­amyloid-beta pathology, depressive symptomatology rendered a weaker although significant association with subjective complaints, in this case, concentration complaints. It should be clarified that we modeled amyloid and tau CSF biomarkers as continuous variables in our analyses and the proportion of ­biomarker-positive amyloid and tau SCD individuals was low. Therefore, subjective complaints are differentially associated with these AD biomarkers but these biomarker levels may be normal and might remain normal over time. One thus needs to be aware that this finding could reflect normal age-related changes and not pathological changes. However, if this differential association of ­amyloid-beta and tau biomarkers with concentration and memory complaints and depressive symptomatology can be replicated in other cohorts, our findings may have clinical implications. One could suggest that people may be able to detect different stages of the biological process of AD through concentration and memory complaints before cognitive decline can be detected with objective neuropsychological tests. This is a first step to disentangle the underpinnings of these different complaints, as well as the different implications of depressive symptomatology in the AD continuum. In addition, such a finding could support the use of certain complaints to enrich research cohorts and clinical trials with certain biomarker profiles (20).

We did not find any significant association between SCD and CVD biomarkers in our cohort, in line with other studies that included similar populations (22,48,49). However, there are studies showing a significant association between SCD and CVD (7,8,50). These diverging results from de Groot et al. (50) could be explained by SCD mostly being related to periventricular WMSA, while our WMSA biomarker is a measure of global burden. The discrepancy with Diaz-Galvan et al. (7) could be related to their wide age range, while our participants were all 70 years old by design. Furthermore, the average age of the cohort in Diaz-Galvan et al. (7) (mean age, 54.6 years) was younger than in our cohort, including age strata where CVD can already be present, but it is more difficult to find amyloid-beta or tau positivity. In Minett et al. (8) WMSA were related to the severity of SCD, rather than the presence of SCD, and complaints were assessed with a memory questionnaire. Furthermore, individuals sought medical help and complaints were elicited by the physician, a different setup than in our study. In addition, we cannot exclude that AD and CVD are 2 additive pathologies, and together they increase the risk for cognitive impairment (46). In our cohort, the presence of both AD pathology and CVD is likely associated with cognitive impairment, which was an exclusion criterion in our current study. This could also explain the diverging results with the findings of de Groot et al. (50), Diaz-Galvan et al. (7), and Minett et al (8).

The present study has some limitations. First, to completely capture the interaction between biomarkers, complaints, and depressive symptomatology throughout the different preclinical phases of AD, our current findings should be complemented with longitudinal studies. To the best of our knowledge, findings from our cross-sectional analyses are the first on the association between these 3 factors and may be useful for the design of future longitudinal studies. Second, Jessen et al. (1) suggested that the complaints should have a duration of 6 months, whereas in the current study, our questions referred to complaints within the last 1 month. This criterion was included by Jessen et al. (1) mostly to increase the ability of SCD to reflect a neurodegenerative disease. It is reassuring that despite referring to the last 1 month in our study, we still captured associations of the complaints with both amyloid-beta and tau biomarkers of AD. Third, we used 2 items from the CPRS to assess SCD. Although the questions used in our study are not specifically developed to assess SCD, these 2 questions align with memory and concentration complaints included in validated scales for SCD such as the ECog (29), which is widely used and is recommended by the international SCD-Initiative. As informed by the SCD-I, around half (47%) of previous studies are based only on 1 item for memory complaints, hence the addition of the concentration complain in our study is an advantage. We further demonstrated that our memory and concentration questions were statistically independent between them, probably representing 2 different cognitive processes likely due to the questionnaire itself distinguishing between concentration failure and memory failure. However, in the future, it would be important to apply more comprehensive questionnaires of memory and concentration complaints, perhaps including other nonmemory domains as well. Finally, we comment on some nonsignificant statistical trends due to their potential clinical interest. To substantiate this approach, we provided effect sizes (or odds ratios) in the respective analyses. Effect sizes are also informative in the context of greater statistical power for the SCD-memory group than for the SCD-concentration group, since we anticipated that it would take larger differences or stronger associations to become statistically significant in the smaller SCD-concentration group. This is because the smaller sample size in the SCD-concentration group provides a lower statistical power than comparisons involving the larger SCD-memory group. To overcome this, we confirmed that none of the large effect sizes (or odds ratios) in the smaller SCD-concentration group were missed to become significant (ie, we ruled out any potential false negative results).

We conclude that in our cohort, depressive symptomatology in SCD can be interpreted as an independent phenomenon of AD and CVD biomarkers. The role of depressive symptomatology may be different depending on the actual stage within the spectrum of preclinical AD (as determined by amyloid-beta and tau positivity). Our findings help to advance the current knowledge on the role of subclinical depressive symptomatology in SCD, a topic that has recently been urged by the international SCD-I (10,25). Moreover, we suggest that subjective complaints of memory and concentration may reflect different stages of AD pathology. This study adds to the still scant literature on the potential association of different subjective cognitive complaints with distinct syndromic and biomarker profiles.

## Supplementary Material

glad216_suppl_Supplementary_Material

## Data Availability

Anonymized data are available from the corresponding author upon reasonable request.
