# Supplementary material for: Biomarkers of Alzheimer’s Disease and Cerebrovascular Disease in Relation to Depressive Symptomatology in Individuals With Subjective Cognitive Decline
Source: J Gerontol A Biol Sci Med Sci. 2023 Sep 14;79(2):glad216. doi: 10.1093/gerona/glad216 (PMC10803123; doi:10.1093/gerona/glad216)
Supplement: glad216_suppl_Supplementary_Material [file glad216_suppl_supplementary_material.docx]

**Supplementary material**

**eTable 1.** Questions used for the operationalization of memory (SCD-memory) and concentration (SCD-concentration) SCD groups.

| SCD-memory: Failing memory. Representing subjective disturbances of recall compared with previous ability. Distinguish from concentration difficulties | |
| --- | --- |
| Control group | 0-1: Memory as usual |
| SCD-memory group | 2-3: Occasional increased lapses of memory |
|  | 4-5: Reports of socially inconvenient or disturbing loss of memory |
|  | 6: Complaints of complete inability to remember |
| SCD-concentration: Concentration difficulties. Representing difficulties in collecting one’s thoughts mounting to incapacitating lack of concentration. Distinguish from failing memory. | |
| Control group | 0-1: No difficulties in concentrating |
| SCD-concentration group | 2-3: Occasional difficulties in collecting one’s thoughts |
|  | 4-5: Difficulties in concentrating and sustaining thought which interfere with reading or conversation |
|  | 6: Incapacitating lack of concentration |

Abbreviations: SCD-memory= Subjective Cognitive Decline in Memory; SCD-concentration= Subjective Cognitive Decline in Concentration

**eTable 2.** Correspondence between items defining SCD-memory and concentration groups in the current study and memory and concentration questions in the ECog questionnaire for SCD [29].

| **SCD-memory (current study)** | **Memory (ECog)** |
| --- | --- |
| Failing memory. Representing subjective disturbances of recall compared with previous ability. Distinguish from concentration difficulties | Difficulties in:  -Remembering a few shopping items without a list.  -Remembering things that happened recently (such as recent outings, events in the news).  -Recalling conversations a few days later.  -Remembering where she/he has placed objects.  -Repeating stories and/or questions.  -Remembering the current date or day of the week.  -Remembering he/she has already told someone something.  -Remembering appointments, meetings, or engagements. |
| **SCD-concentration (current study)** | **Attention/Executive function (ECog)** |
| Concentration difficulties. Representing difficulties in collecting one’s thoughts mounting to incapacitating lack of concentration. Distinguish from failing memory. | Difficulties in:  -The ability to do two things at once.  - Returning to a task after being interrupted.  -The ability to concentrate on a task without being distracted by external things in the environment.  -Cooking or working and talking at the same time.  -Planning the sequence of stops on a shopping trip.  -The ability to anticipate weather changes and plan accordingly.  -Developing a schedule in advance of anticipated events.  -Thinking ahead.  -Thinking things through before acting.  -Keeping living and work space organized.  -Balancing the checkbook without error.  -Keeping financial records organized.  -Prioritizing tasks by importance.  -Using an organized strategy to manage a medication schedule.  -Keeping mail and papers organized. |

Abbreviations: SCD-memory= Subjective Cognitive Decline in Memory; SCD-concentration= Subjective Cognitive Decline in Concentration ; ECog= Everyday cognition.

**eFigure 1. Study selection flow
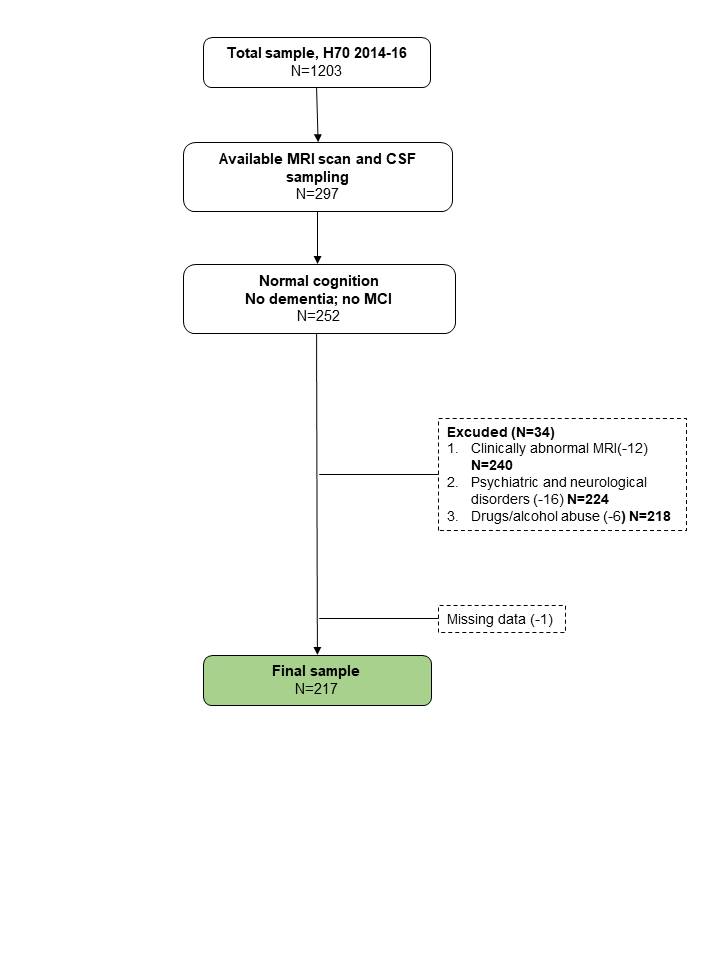
**Study selection flow diagram with the number of participants excluded from the study at each step, showing the final study sample. Abbreviations: MRI= magnetic resonance imaging; CSF= cerebrospinal fluid; MCI= Mild cognitive impairment.

**eFigure 2.** Depressive symptomatology - Distribution of MADRS scores.

**
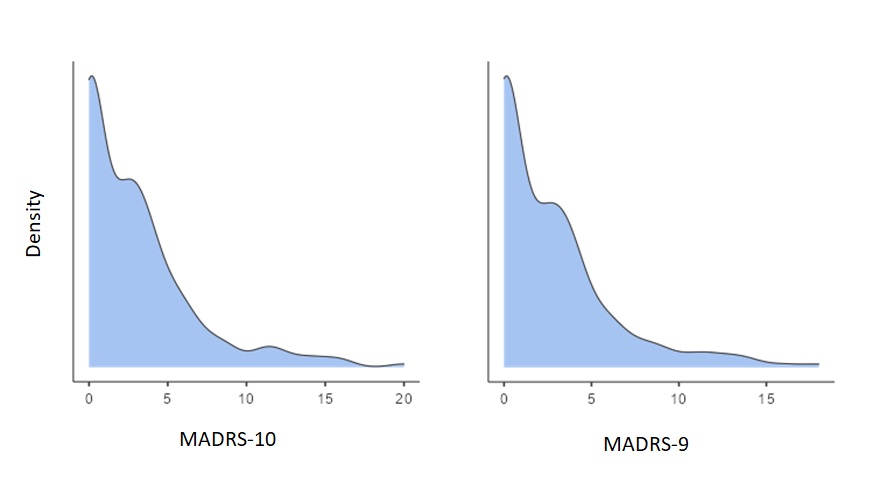
**

Distribution of total MADRS-10 and MADRS-9 scores in the study. MADRS scores on the x-axis and density on the y-axis. Abbreviation: MADRS-10= The Montgomery-Åsberg Depression Rating Scale 10-items; MADRS-9= The Montgomery-Åsberg Depression Rating Scale with the concentration item excluded.

**Logistic regression analyses:**

***Partial association of CSF biomarkers, hyperintense WMSA, and depressive symptomatology with SCD-memory***

Binary logistic regression was conducted including SCD-memory as the criterion variable (SCD-memory *vs.* controls), and MADRS-9, Aβ42/40, p-tau, and hyperintense WMSA as the predictors. The model was significant (χ^2^(2) = 8.550, *p* =.014, *R*^2^ =.054 (Nagelkerke), showing that higher MADRS-9 scores (*B=* .108, Wald = 5.042, SE = .048, *p* = .025, OR = 1.114) and higher p-tau (*B=* .133, Wald = 3.744, SE =.069, *p* =.053, OR = 1.142) significantly predicted SCD-memory. The SCD-memory group had higher p-tau levels and MADRS-9 scores. However, Aβ42/40(*B=* -.032, Wald = .000, SE = 2.094, *p* =.988, OR = .968) and hyperintense WMSA (*B=* 2.745, Wald =.186, SE = 6.365, *p* =.666, OR = 15.559) were not significantly associated with SCD-memory.

***Partial association of CSF biomarkers, hyperintense WMSA, and depressive symptomatology with SCD-concentration***

We performed similar models for SCD-concentration. SCD-concentration was included as the criterion variable (SCD-concentration *vs.* controls), and MADRS-9, Aβ42/40, and p-tau as well as hyperintense WMSA as the predictors. The model was significant (χ^2^(2) = 21.422, *p* <.001, *R*^2^ =.274 (Nagelkerke). Aβ42/40 (*B=* -15.302, Wald = 11.813, SE = 4.653, *p* = .001, OR = .000) was the main predictor of SCD-concentration, followed by MADRS-9 (*B=* .201, Wald = 7.045, SE = .076, *p* = .008, OR = 1.223). The SCD-concentration group had lower levels of Aβ42/40 and MADRS-9 scores. In contrast, p-tau (*B= -*.001, Wald =.000, SE =.132, *p* =.991, OR =.999), and hyperintense WMSA (*B=*9.013, Wald =.596, SE = 11.675, *p* =.440, OR = 8206.437) were not significantly associated with SCD-concentration.
